# *Artemisia annua* L. Aqueous Extract Promotes Intestine Immunity and Antioxidant Function in Broilers

**DOI:** 10.3389/fvets.2022.934021

**Published:** 2022-07-08

**Authors:** Shiwei Guo, Jiaxin Ma, Yuanyuan Xing, Lulu Shi, Linghui Zhang, Yuanqing Xu, Xiao Jin, Sumei Yan, Binlin Shi

**Affiliations:** College of Animal Science, Inner Mongolia Agricultural University, Hohhot, China

**Keywords:** feed additive, plant-based ingredients, *Artemisia annua L*., broiler health, gut, immune status, antioxidation

## Abstract

This study was conducted to investigate the effects of *Artemisia annua* L. aqueous extract (AAE) on intestinal immune and antioxidative function of broilers. A total of 200 one-day-old Arbor Acre broilers were randomly allotted into five dietary treatment groups, with five replicates per treatment and eight broilers per replicate. The five treatment diets were formulated by adding, respectively, 0 (control group), 0.5, 1.0, 1.5, and 2.0 g/kg AAE in the basal diet. The results showed that dietary inclusion of AAE quadratically decreased interleukin (IL)-1β content, linearly decreased IL-6 content in the small intestine through regulating the nuclear factor-kappa B signal pathway, and quadratically increased immunoglobulin (Ig)M and sIgA content in ileum and jejunum. Besides, there was a quadratic decrease in the gene expression of *IL-1*β, *IL-6*, and toll like receptor 4 (*TLR4)* in ileum on day 21, and the gene expression of *IL-6* and *TLR4* in duodenum on day 42, thereby improving small intestinal immune function in broilers. Additionally, dietary inclusion of AAE improves antioxidative function through the nuclear factor-erythroid 2-related factor 2 (Nrf2) signal pathway in the small intestinal mucosa of broilers, especially, quadratically increased catalase (CAT) and superoxidase dismutase activity in ileum, and total antioxidant capacity and glutathione peroxidase activity in duodenum, and quadratically decreased malondialdehyde concentration in ileum, besides, linearly increased *heme oxygenase-1* and *Nrf2* gene expression in jejunum and ileum on day 42, quadratically increased *CAT* gene expression in the small intestine. Furthermore, regression analyses of the above parameters showed that the optimal dose range of AAE in the diet of broilers was 1.12–1.38 g/kg.

## Introduction

Recently, many countries and organizations have formulated regulations and systems that prohibit the use of feed antibiotics (1–3). Thus, it has prompted people to search for natural substitutes of antibiotic, such as plant extracts (4). Liu et al. (5) found that dietary plant extract (natural capsicum extracts) improved growth performance, immune and antioxidative functions of broilers, and suggested that the extract could be used as an effective alternative to antibiotics in broilers. It is worth noting that *Artemisia* plant extracts are rich in a variety of bioactive constituents, which can promote the growth, immune function, and antioxidant capacity of broilers (6–8).

*Artemisia annua* L. (*A. annua*), a kind of *Artemisia* plant, is well-known for its medicinal properties and wide distribution around the world (9–11). Recently, artemisinin, an antimalarial component of *A. annua* studied by Tu, has attracted wide attention worldwide (12). *A. annua* has multiple properties, such as anticancer (13), anti-malarial (14), anti-inflammatory (15), and antioxidant (11), which are related to its rich bioactive constituents, including polysaccharides (16), polyphenols (17), and flavonoids (18). It was reported that dietary inclusion of dried *A. annua* leaves was used for the coccidiosis treatment and growth advancement in broilers (19, 20). Coincidentally, *A. annua* leaves had significant free radical scavenging ability and antioxidant ability *in vitro* (21). In addition, dietary *A. annua* leaves positively influenced the plasma antioxidant indexes and significantly decreased the concentration of egg yolk cholesterol, with no negative effect on the egg weight and laying rate of hens (22). Wan et al. (23) also reported that dietary enzymatically treated *A. annua* could improve meat quality, antioxidant capacity, and energy status of breast muscle in broilers. Moreover, dietary enzymatically treated *A. annua* supplementation enhanced intestinal immunity and antioxidant capacity of weaned piglets (24, 25). At high temperatures, dietary supplementation with enzymatically treated *A. annua* improved intestinal sIgA and IgG content, and antioxidant capacity of broilers (26). Previous reports show that sesquiterpenoids from the aerial parts of *A. annua* had an inhibitory activity against the production of inflammatory cytokines (PGE2, NO, IL-6, and TNF-α) in lipopolysaccharide (LPS)-induced RAW 264.7 macrophages (15). Furthermore, aqueous and alcoholic extracts of *A. annua* improved insulin resistance *via* decreasing TNF-alpha and IL-6 in high-fat diet/streptozotocin-induced diabetic mice (27). Our previous study found that *Artemisia argyi* and *Artemisia ordosica* aqueous extract could improve the immune and antioxidant status of LPS-induced broilers (28, 29). Given these features, the present study used water as a solvent to extract the bioactive components of *A. annua*, and aimed to investigate the effects of *A. annua* aqueous extract on intestinal immune function, and antioxidant capability in broilers.

## Materials and Methods

### Preparation of *Artemisia annua* L. Aqueous Extract

Fresh *A. annua* was harvested from Hohhot (Inner Mongolia, China) in July. Raw materials were washed with distilled water and dried at room temperature. The dried plant was extracted in hot distilled water at 80°C for 6 h, and the supernatant of the extract liquor was collected, and the filtrate was concentrated using a rotary vacuum evaporator (RE-5298, Shanghai Yarong Biochemical Instrument Factory, Shanghai, China) at 70°C, and then was freeze-dried (ALPHA1-2LD plus, Christ, Germany) to prepare the powder, and stored at −20°C. Using this preparation process, 250 g of *A. annua* aqueous extract can be obtained per kilogram of dried *A. annua* raw material. Moreover, the total phenolic and flavonoid contents were, respectively, 39.58 mg GAE/g and 7.04 mg RE/g.

### Birds, Experimental Design, and Diets

A total of 200 one-day-old Arbor Acres broilers were purchased from a commercial hatchery in Hohhot, Inner Mongolia, China. The birds were randomly divided into five treatment groups with five replicates of eight birds each. These five treatment diets were formulated by adding, respectively, 0, 0.5, 1.0, 1.5, and 2.0 g/kg AAE into the basal diet (Table 1). The feeding experiment lasted for 42 days, divided into the starter period (days 1–21) and the finisher period (days 22–42). Diets were formulated to meet the nutritional recommendations of the Feeding Standard of Chicken, China (NY/T 33-2004) (30) (Table 1). Experimental diets and water were available *ad libitum*. According to the method reported by De Oliveira and Lara (31), the chicken houses were illuminated by LED lights that provide 30–40 lux of light intensity. The lighting scheme included 23 h lighting (L):1 h darkness (D) (23L:1D, days 0–3), 10 L:14 D (days 4–21), 14 L:10 D (days 22–28), 18 L:6 D (days 29–35), and 23 L:1 D (days 36–42). The temperature of the experimental room was set at 32–34°C for the first 3 days and then gradually reduced by 3°C every week, and a final temperature of 21°C was reached. The relative humidity was maintained at about 55 ± 5%. The vaccination procedure was conducted as follows: the broilers were vaccinated with Newcastle disease and infectious bronchitis combined vaccine on days 7 and 28, Newcastle disease, infectious bronchitis, and avian influenza triple vaccine on day 10, and infectious bursal disease vaccine on days 14 and 20. All animal experiments were performed following the national standard Guideline for Ethical Review of Animal Welfare (GB/T 35892-2018).

**Table 1:** Composition and nutrient levels of the basal diet (as-fed basis), %.

**Items**	**1 to 21 days of age**	**22 to 42 days of age**
**Ingredients**		
Corn	52.50	58.80
Soybean meal	40.00	33.80
Soybean oil	3.00	3.00
Dicalcium phosphate	1.90	1.80
Limestone	1.08	1.22
Salt	0.37	0.37
Lysine	0.05	0.03
Methionine	0.19	0.07
Premix^a^	0.80	0.80
Choline	0.11	0.11
Total	100.0	100.0
**Nutrient levels** ^**b**^		
Metabolic energy (MJ/kg)	12.42	12.62
Crude protein	21.77	19.65
Calcium	1.00	1.02
Available phosphorus	0.44	0.42
Lysine	1.34	1.15
Methionine	0.55	0.40
Cystine	0.40	0.36

^a^*Premix provided the following per kilogram of diet: vitamin A 9,000 IU, vitamin D_3_ 3,000 IU, vitamin E 26 mg, vitamin K_3_ 1.20 mg, vitamin B_1_ 3.00 mg, vitamin B_2_ 8.00 mg, vitamin B_6_ 4.40 mg, vitamin B_12_ 0.012 mg, nicotinic acid 45 mg, folic acid 0.75 mg, biotin 0.20 mg, choline 1100 mg, calcium pantothenate 15 mg, Fe 100 mg, Cu 10 mg, Zn 108 mg, Mn 120 mg, I 1.5 mg, Se 0.35 mg*.

*^b^Crude protein was the measured value, while others were all calculated values*.

### Preparation of Intestinal Homogenate

On days 21 and 42, one bird was randomly selected from each replicate and then euthanized by exsanguination. The abdominal cavity of the broiler was opened on ice, and the intestinal tract was taken out, and then the duodenum, jejunum, and ileum were separated and stored in a centrifuge tube at −20°C for further analysis, which was conducted according to the following procedure.

The intestinal pieces were homogenized with a hand-held homogenizer (FA6/10, FLUKO, Shanghai, China) at 4°C in ice-cold 0.9% sodium chloride solution (wt/vol, 1:9) and then centrifuged at 4,000 × *g* for 15 min at 4°C. The supernatant was collected for follow-up analysis. Coomassie brilliant blue assay was used to determine the protein of the homogenate according to the instructions of the commercial kits (Nanjing Jiancheng Institute of Bioengineering, Nanjing, China).

### Determination of Intestinal Immunity Function

Interleukin-1 beta (IL-1β), interleukin-6 (IL-6), immunoglobulin G (IgG), immunoglobulin M (IgM), and secretory immunoglobulin A (sIgA) concentrations were analyzed using ELISA kits (Quanzhou Ruixin Biological Technology Co., Ltd. Fujian, China) following the manufacturer's instructions.

### Determination of Intestinal Antioxidant Function

The total antioxidant capacity (TAC), the activity of total superoxide dismutase (SOD), glutathione peroxidase (GPx), and catalase (CAT), and the concentration of malondialdehyde (MDA) in the intestine were determined by a spectrophotometric method according to the instructions of the commercial kits (Nanjing Jiancheng Institute of Bioengineering, Nanjing, China). The activity of SOD, GPx, and CAT in intestinal mucosa was expressed as activity unit per milligram of tissue protein (unit/mg protein). The concentration of MDA was expressed as nanomole per milligram of tissue protein (nmol/mg protein). TAC was expressed as micromole (μmol) Trolox equivalent per gram protein of homogenate (μmol/g protein).

### Total RNA Extraction and Reverse Transcription

Total RNA from intestinal samples was obtained using Trizol reagent (TaKaRa Biotechnology Co. Ltd, Dalian, China). The purity and quantity of the total RNA were assessed with a spectrophotometer (Pultton P200CM, San Jose, CA, USA). Subsequently, the total RNA was treated with DNase I (TaKaRa) to remove DNA. Total RNA was reverse transcribed to cDNA on LifeECO (TC-96/G/H(b)C, BIOER, Hangzhou, China) using TB® Green qPCR method with a Prime Script™ RT reagent kit with gDNA Eraser (TaKaRa Biotechnology Co. Ltd., Dalian, China). The reactions were incubated for 15 min at 37°C, followed by 5 s at 85°C.

### Quantitative Real-Time PCR

Real-time PCR was performed using the QuantStudio®5 real-time PCR Design & Analysis system (LightCycler® 480 II, Roche Diagnostics, USA) with a TB® Premix Ex Taq™ Kit (Takara Biotechnology Co. Ltd., Dalian, China). The reactions were 95°C for 30 s (hold stage), followed by 40 cycles of 95°C for 5 s, 60°C for 30 s, and 72°C for 20 s (PCR stage), then 95°C for 15 s, 60°C for 1 min, 95°C for 15 s (melt-curve stage). All samples were run in duplicate in 20 μl reaction volume and melt curve analysis was performed to validate the specificity of the PCR-amplified product. The mRNA expression of each gene was normalized to that of β-actin. The fold change relative to the control group was analyzed according to the 2^−ΔΔCT^ method (32). The specific sequences of primers are listed in Table 2.

**Table 2:** Primer sequences and parameter.

**Genes**	**Gene Bank** **no**.	**Primer sequences, 5'-3'**	**Length, bp**
*β-actin*	NM_205518	F. GCCAACAGAGAGAAGATGACAC	118
		R. GTAACACCATCACCAGAGTCCA	
*IL-1β*	NM_204524	F. CAGCCTCAGCGAAGAGACCTT R. ACTGTGGTGTGCTCAGAATCC	84
*IL-6*	HM179640	F. AAATCCCTCCTCGCCAATCT R. CCCTCACGGTCTTCTCCATAAA	106
*TLR4*	NM_001030693	F. TTCAGAACGGACTCTTGAGTGG	131
		R. CAACCGAATAGTGGTGACGTTG	
*NF-κB /p65*	D13721	F. CAGCCCATCTATGACAACCG R. CAGCCCAGAAACGAACCTC	151
*CAT*	NM_001031215.1	F. GTTGGCGGTAGGAGTCTGGTCT R. GTGGTCAAGGCATCTGGCTTCTG	182
*SOD*	NM_205064.1	F. TTGTCTGATGGAGATCATGGCTTC R. TGCTTGCCTTCAGGATTAAAGTGA	98
*GPx*	NM_001163245.1	F. CAAAGTTGCGGTCAGTGGA R. AGAGTCCCAGGCCTTTACTACTTTC	136
*HO-1*	HM237181.1	F. GGTCCCGAATGAATGCCCTTG	138
		R. ACCGTTCTCCTGGCTCTTGG	
*Keap1*	XM_015274015.1	F. TGCCCCTGTGGTCAAAGTG	104
		R. GGTTCGGTTACCGTCCTGC	
*Nrf2*	NM_205117.1	F. GATGTCACCCTGCCCTTAG	215
		R. CTGCCACCATGTTATTCC	

*β-Actin, beta-actin; IL-1β, interleukin 1 beta; IL-6, interleukin 6; TLR4, toll like receptor 4; NF-κB /p65, nuclear factor kappa B/p65; CAT, catalase; SOD, total superoxide dismutase; GPx, glutathione peroxidase; HO-1, heme oxygenase-1; Keap1, Kelch-like ECH-associated protein-1; Nrf2, nuclear factor-erythroid 2-related factor 2; F, forward primer; R, reverse primer*.

### Statistical Analysis

All collected data were first processed by Microsoft Excel 2019, and then analyzed by one-way ANOVA using statistical analysis software SAS 9.2 (SAS Institute Inc., Cary, NC, USA). The individual broiler was an experimental unit for all the data. Differences among treatments were evaluated by Duncan's multiple range test. Meanwhile, regression analysis was conducted to evaluate the linear and quadratic effects of the increasing levels of AAE on the various indexes. Quadratic regressions (Y = aX^2^ + bX + c) were fitted to the responses of the dependent variables to dietary AAE supplemented levels. The extremum response for AAE was defined as AAE = - b/ (2 × a). The results are expressed as mean ± standard deviation. The probability value of *p* < 0.05 was considered to be statistically significant, whereas the probability value of 0.05 ≤ *p* < 0.10 was considered as a tendency.

## Results

### Small Intestinal Cytokine Content

The small intestinal cytokine contents are summarized in Table 3. On day 21, compared with the control group, 1.0–2.0 g/kg AAE groups had lower duodenal IL-1β content (*p* < 0.01), 1.5 and 2.0 g/kg AAE groups exhibited lower jejunal IL-6 content (*p* < 0.05), and all AAE groups exhibited lower jejunal IL-1 and ileal IL-6 content (*p* < 0.05). Moreover, with the increase of AAE dose, the duodenal IL-1β and IL-6, and jejunal IL-6 content had a linear reduction effect (*p* < 0.01), and the content of IL-1β in the three parts of small intestine and the ileal IL-6 content had a quadratic reduction effect (*p* < 0.01, *p* < 0.05, *p* < 0.10, *p* < 0.01). On day 42, compared with the control group, the jejunal IL-1β and IL-6 content of 1.0–2.0 g/kg in the AAE group was remarkably reduced (*p* < 0.01). All AAE groups had lower duodenal IL-6 content (*p* < 0.05), and the ileal IL-6 content of 1.5 g/kg in the AAE group had significantly decreased (*p* < 0.05). Besides, the duodenal and jejunal IL-1β content, and the jejunal and ileal IL-6 content showed a linear reduction effect (*p* < 0.05); besides, the jejunal and ileal IL-1β content, and the duodenal and jejunal IL-6 content showed a quadratic reduction effect (*p* < 0.05). According to a quadratic regression analysis, the minimum response for jejunum IL-1β content on day 42 was observed at 1.8070 g/kg. Besides, for IL-6 content on day 42 in the duodenum, the optimum level was 1.3868 g/kg (Table 4).

**Table 3:** Effect of AAE on cytokine content in small intestine of broilers.

**Items**	**AAE supplemental level, g/kg**					***p*** **-value**		
	**0**	**0.5**	**1.0**	**1.5**	**2.0**	**ANOVA**	**Linear**	**Quadratic**
**21 d**								
**IL-1β** **pg/mg prot**.								
Duodenum	23.91 ± 1.53^a^	21.41 ± 2.17^a^	17.31 ± 3.09^b^	14.92 ± 2.70^b^	10.58 ± 0.94^c^	<0.001	<0.001	<0.001
Jejunum	15.25 ± 2.40^a^	10.59 ± 1.64^b^	11.08 ± 2.08^b^	11.06 ± 0.66^b^	11.74 ± 1.22^b^	0.022	0.198	0.015
Ileum	19.49 ± 3.68	16.62 ± 3.27	14.61 ± 2.56	14.52 ± 3.89	16.62 ± 2.39	0.301	0.147	0.074
**IL-6 pg/mg prot**.								
Duodenum	0.66 ± 0.12	0.70 ± 0.07	0.54 ± 0.13	0.54 ± 0.14	0.42 ± 0.09	0.118	0.010	0.036
Jejunum	0.47 ± 0.12^a^	0.44 ± 0.06^a^	0.44 ± 0.08^a^	0.28 ± 0.08^b^	0.28 ± 0.05^b^	0.013	0.002	0.006
Ileum	0.16 ± 0.03^a^	0.09 ± 0.01^b^	0.10 ± 0.01^b^	0.06 ± 0.01^c^	0.11 ± 0.01^b^	<0.001	0.015	0.001
**42 d**								
**IL-1β** **pg/mg prot**.								
Duodenum	18.59 ± 0.60	18.75 ± 1.04	17.91 ± 1.53	17.91 ± 0.62	16.41 ± 1.77	0.125	0.015	0.031
Jejunum	24.08 ± 1.75^a^	22.35 ± 2.76^a^	18.23 ± 2.73^b^	17.12 ± 0.67^b^	17.76 ± 1.30^b^	<0.001	<0.001	<0.001
Ileum	15.84 ± 0.82	13.16 ± 3.97	11.86 ± 1.23	13.25 ± 0.86	13.54 ± 1.23	0.115	0.158	0.030
**IL-6 pg/mg prot**.								
Duodenum	0.81 ± 0.04^a^	0.59 ± 0.08^b^	0.55 ± 0.10^b^	0.52 ± 0.13^b^	0.56 ± 0.03^b^	0.030	0.015	0.004
Jejunum	0.46 ± 0.12^a^	0.33 ± 0.09^ab^	0.26 ± 0.06^b^	0.19 ± 0.02^b^	0.18 ± 0.03^b^	0.008	0.001	0.001
Ileum	0.11 ± 0.02^a^	0.10 ± 0.00^a^	0.10 ± 0.01^ab^	0.06 ± 0.01^b^	0.08 ± 0.01^ab^	0.036	0.008	0.026

*AAE, Artemisia annua L. aqueous extract; IL-1β, interleukin 1 beta; IL-6, interleukin 6. ^a−c^Means within a row with different letters differ significantly (p < 0.05), whereas the probability value of 0.05 ≤ p < 0.10 was considered as a tendency. Each value is shown as mean ± SD (Data are means for five replicates of eight birds per replicate)*.

**Table 4:** Estimation of the extremum response for dietary AAE levels based on quadratic regressions in broilers.

**Dependent variables**	**Regression equation**	***R* ^2^**	***p***	**Optimum Dietary AAE, g/kg**
**21 d**				
IgG (Duodenum), μg/mg prot.	Y = −11.563X^2^ + 25.972X + 45.159	0.9394	0.015	1.1231
IgM (Ileum), μg/mg prot.	Y = −3.24X^2^ + 8.12X + 18.348	0.9125	0.004	1.2531
CAT (Ileum), U/mg prot.	Y = −0.3086X^2^ + 0.8091X + 0.7597	0.9907	0.006	1.3109
SOD (Ileum), U/mg prot.	Y = −52.2X^2^ + 139.18X + 192.58	0.9223	0.021	1.3331
GPx (Duodenum), U/mg prot.	Y = −2.9429X^2^ + 6.2497X + 5.6966	0.9971	0.039	1.0618
*Keap1* (Ileum)	Y = 0.1886X^2^-0.5091X + 0.9963	0.8975	0.004	1.3497
**42 d**				
IL-1β (Jejunum), pg/mg prot.	Y = 2.2143X^2^-8.0026X + 24.589	0.9391	<.001	1.8070
IL-6 (Duodenum), pg/mg prot.	Y = 0.1514X^2^-0.4169X + 0.7957	0.9639	0.004	1.3868
IgM (Jejunum), μg/mg prot.	Y = −3.5771X^2^ + 8.7623X + 18.737	0.8896	0.055	1.2248
sIgA (Ileum), ng/mg prot.	Y = −11.057X^2^ + 29.974X + 35.729	0.9811	0.001	1.3554
*TLR4* (Duodenum)	Y = 0.2371X^2^-0.6163X + 1.0006	0.9982	0.020	1.2997
CAT (Ileum), U/mg prot.	Y = −0.2914X^2^ + 0.7469X + 0.9823	0.9637	0.039	1.2816
TAC (Duodenum), umol/g prot.	Y = −28.057X^2^ + 74.594X + 104.49	0.9900	0.005	1.3293
TAC (Jejunum), umol/g prot.	Y = −22.029X^2^ + 52.197X + 121.67	0.8921	0.002	1.1847
*CAT* (Ileum)	Y = −0.6571X^2^ + 1.5263X + 0.9074	0.8575	0.001	1.1614

*AAE, Artemisia annua L. aqueous extract; TAC, total antioxidant capacity; CAT, catalase; SOD, total superoxide dismutase; GPx, glutathione peroxidase; IgG, immunoglobulin G; IgM, immunoglobulin M; sIgA, secretory immunoglobulin A. IL-1β, interleukin 1 beta; IL-6, interleukin 6; TLR4, toll like receptor 4; Keap1, Kelch-like ECH-associated protein-1; Extremum was the maximum or minimum response to dietary AAE levels according to each regression equation (g/kg); R^2^, determination coefficient; p, P-value of quadratic effect; Y was the dependent viable; X was the dietary AAE level (g/kg)*.

### Small Intestinal Immunoglobulin Content

As described in Table 5, on day 21, compared with the control group, the AAE groups with the values of 1.0 and 1.5 g/kg tended to increase the duodenum IgG content (*p* < 0.10); moreover, the dietary AAE groups with the values of 1.0 and 1.5 g/kg significantly increased the duodenal and ileal IgM content (*p* < 0.05). Besides, with the increase of AAE dose, the content of duodenal IgG, IgM, and sIgA, and ileal IgM showed a quadratic increased effect (*p* < 0.05, *p* < 0.01, *p* < 0.10, *p* < 0.01). According to a quadratic regression analysis, the maximum response for the duodenum IgG content and ileum IgM content were observed at levels of 1.1231 and 1.2531 g/kg in the AAE group, respectively (Table 4). On day 42, compared with the control group, the AAE groups with the values of 1.0–2.0 g/kg significantly increased the ileal IgG content (*p* < 0.05); all the AAE groups remarkably increased the ileal content of IgM and sIgA (*p* < 0.05). Moreover, the content of ileal IgG, IgM, sIgA, and jejunal IgM and sIgA showed a quadratic increased effect (*p* < 0.01, *p* < 0.01, *p* < 0.01, *p* < 0.10, *p* < 0.10). As shown in Table 4, for jejunum IgM content, the optimal AAE level was 1.2248 g/kg; besides, for ileum sIgA content, the optimum level was 1.3554 g/kg.

**Table 5:** Effect of AAE on immunoglobulin content in small intestine of broilers.

**Items**	**AAE supplemental level, g/kg**					***p*** **-value**		
	**0**	**0.5**	**1.0**	**1.5**	**2.0**	**ANOVA**	**Linear**	**Quadratic**
**21 d**								
**IgG ug/mg prot**.								
Duodenum	46.16 ± 2.26^b^	52.94 ± 8.35^ab^	60.50 ± 7.52^a^	59.17 ± 9.26^a^	50.16 ± 5.08^ab^	0.071	0.438	0.015
Jejunum	48.46 ± 4.30	53.91 ± 9.43	62.58 ± 8.82	54.85 ± 11.77	50.96 ± 7.73	0.276	0.596	0.122
Ileum	60.39 ± 9.46	63.00 ± 4.35	70.19 ± 9.14	64.55 ± 5.26	63.41 ± 6.01	0.350	0.522	0.265
**IgM ug/mg prot**.								
Duodenum	17.88 ± 0.96^bc^	20.44 ± 0.57^ab^	24.38 ± 2.42^a^	24.62 ± 4.94^a^	13.92 ± 2.47^c^	<0.001	0.635	0.001
Jejunum	19.40 ± 1.33	19.78 ± 1.95	25.10 ± 4.09	20.87 ± 3.13	20.89 ± 3.99	0.179	0.450	0.295
Ileum	18.69 ± 1.43^b^	20.67 ± 2.44^ab^	23.96 ± 0.55^a^	23.19 ± 3.16^a^	21.53 ± 1.76^ab^	0.020	0.028	0.004
**sIgA ng/mg prot**.								
Duodenum	47.60 ± 4.48	49.15 ± 4.45	56.24 ± 8.89	60.63 ± 9.92	50.35 ± 4.62	0.102	0.133	0.082
Jejunum	48.37 ± 3.40	50.65 ± 6.96	57.26 ± 8.31	59.10 ± 1.80	50.98 ± 8.80	0.256	0.379	0.131
Ileum	56.34 ± 5.69	58.45 ± 5.62	57.02 ± 8.54	61.52 ± 4.51	62.81 ± 4.69	0.584	0.111	0.279
**42 d**								
**IgG ug/mg prot**.								
Duodenum	60.73 ± 7.71	63.55 ± 10.55	69.39 ± 8.51	73.60 ± 8.36	61.12 ± 9.77	0.239	0.489	0.152
Jejunum	66.96 ± 5.76	77.39 ± 6.60	80.72 ± 10.15	75.61 ± 11.85	75.27 ± 10.28	0.346	0.333	0.144
Ileum	39.53 ± 3.46^b^	49.42 ± 8.57^ab^	51.75 ± 8.53^a^	57.21 ± 9.09^a^	52.50 ± 4.47^a^	0.019	0.005	0.003
**IgM ug/mg prot**.								
Duodenum	18.10 ± 3.64	19.09 ± 2.24	22.03 ± 2.89	21.64 ± 1.91	20.01 ± 2.64	0.303	0.274	0.117
Jejunum	19.12 ± 1.31	21.15 ± 1.71	24.85 ± 4.95	23.67 ± 4.78	21.88 ± 1.70	0.180	0.161	0.055
Ileum	12.38 ± 1.32^b^	17.73 ± 1.41^a^	18.46 ± 0.89^a^	17.80 ± 3.13^a^	18.86 ± 1.80^a^	0.010	0.008	0.004
**sIgA ng/mg prot**.								
Duodenum	52.49 ± 3.06	55.44 ± 5.52	60.56 ± 8.33	63.15 ± 7.49	55.44 ± 7.66	0.345	0.393	0.156
Jejunum	56.09 ± 4.30	62.16 ± 8.46	69.31 ± 9.18	64.30 ± 5.96	66.82 ± 5.61	0.139	0.051	0.051
Ileum	35.90 ± 4.33^b^	47.08 ± 9.65^a^	56.24 ± 4.66^a^	54.56 ± 6.28^a^	51.81 ± 6.01^a^	0.008	0.007	0.001

*AAE, Artemisia annua L. aqueous extract; IgG, immunoglobulin G; IgM, immunoglobulin M; sIgA, secretory immunoglobulin A*.

*^a−c^Means within a row with different letters differ significantly (p < 0.05), whereas the probability value of 0.05 ≤ p < 0.10 was considered as a tendency*.

*Each value is shown as mean ± SD (Data are means for five replicates of eight birds per replicate)*.

### Small Intestine Antioxidant Index

As illustrated in Table 6, on day 21, compared with the control group, the AAE group with a value of 1.5 g/kg tended to increase the duodenal CAT activity and TAC capacity (*p* < 0.10), the AAE group with the values of 1.0 and 1.5 g/kg significantly increased ileal CAT activity (*p* < 0.05), and the AAE group with a value 1.5 g/kg remarkably increased ileal SOD activity (*p* < 0.10). And MDA concentration in duodenum of 0.5 and 2.0 g/kg AAE groups, jejunum of 0.5–1.5 g/kg AAE groups, and ileum of 1.0 and 1.5 g/kg AAE groups was significantly decreased (*p* < 0.05). Moreover, with the increase of AAE dose, the jejunal CAT activity showed a significant linear increased effect (*p* < 0.05), and the activity of ileal CAT and SOD, duodenal SOD, and GPx showed a significant quadratic increased effect (*p* < 0.05). The levels of jejunal TAC showed a linear upward trend (*p* < 0.10), and the levels of duodenal TAC showed a quadratic increased effect (*p* < 0.05), the duodenal and jejunal MDA concentration showed a linear reduction effect (*p* < 0.01), and the ileal MDA concentration showed a quadratic reduction effect (*p* < 0.01). Moreover, the results from a quadratic regression analysis showed that the optimal AAE levels that maximized CAT and SOD activity of the ileum were 1.3109 and 1.3331 g/kg, respectively. Besides, for duodenum GPx activity, the optimum level was 1.0618 g/kg (Table 4). On day 42, compared with the control group, the AAE groups with the values of 1.0 and 1.5 g/kg tended to increase the duodenal CAT activity (*p* < 0.10); however, SOD activity in duodenum of AAE groups with the values of 1.0–2.0 g/kg, jejunum of AAE groups with the values of 1.0 and 1.5 g/kg, and ileum of AAE groups with the values of 1.5 and 2.0 g/kg was increased (*p* < 0.10, *p* < 0.05, *p* < 0.01). And the duodenal GPx activity in the AAE group with a value of 1.5 g/kg had an upward trend (*p* < 0.10). Dietary AAE groups significantly increased the small intestine TAC capacity (*p* < 0.05). MDA concentration in jejunum of AAE groups with the values of 1.0 and 1.5 g/kg, and ileum of AAE group with a value of 1.0 g/kg was compared with the control group (*p* < 0.05, *p* < 0.10). Besides, with the increase of AAE dose, the activity of duodenal and ileal SOD, and jejunal GPx showed a linear increased effect (*p* < 0.01, *p* < 0.01, *p* < 0.10), and the activity of duodenal CAT and GPx, jejunal SOD, and ileal CAT, SOD, and GPx showed a quadratic increased effect (*p* < 0.10, *p* < 0.10, *p* < 0.05, *p* < 0.05, *p* < 0.01, *p* < 0.10). With the increase of AAE dose, the ileal TAC showed a linear increased effect (*p* < 0.01), and the duodenal and jejunal TAC showed a significant quadratic increase effect (*p* < 0.01), and the MDA concentration of jejunum and ileum showed a significant quadratic reduction effect (*p* < 0.05). As shown in Table 4, for ileum CAT activity, the optimal AAE levels was 1.2816 g/kg; besides, for duodenum and jejunum TAC, the optimum values were 1.3293 and 1.1847 g/kg, respectively.

**Table 6:** Effect of AAE on small intestine antioxidant indexes in broilers.

**Items**	**AAE supplemental level, g/kg**					***p*** **-value**		
	**0**	**0.5**	**1.0**	**1.5**	**2.0**		**ANOVA**	**Linear**	**Quadratic**
**21 d**								
**CAT U/mg prot**.								
Duodenum	0.95 ± 0.06^b^	1.01 ± 0.09^b^	1.12 ± 0.27^ab^	1.40 ± 0.32^a^	1.04 ± 0.15^b^		0.070	0.172	0.133
Jejunum	0.82 ± 0.10	0.95 ± 0.27	1.27 ± 0.20	1.01 ± 0.23	1.28 ± 0.28		0.148	0.040	0.116
Ileum	0.77 ± 0.14^b^	1.07 ± 0.17^ab^	1.25 ± 0.30^a^	1.31 ± 0.22^a^	1.13 ± 0.14^ab^		0.045	0.029	0.006
**SOD U/mg prot**.								
Duodenum	331.20 ± 45.19	415.11 ± 47.13	425.07 ± 72.47	362.68 ± 9.03	317.62 ± 50.96		0.177	0.441	0.048
Jejunum	219.09 ± 27.01	243.29 ± 64.79	280.59 ± 62.57	264.02 ± 54.55	250.78 ± 69.82		0.768	0.477	0.426
Ileum	186.96 ± 19.21^b^	264.68 ± 38.31^ab^	266.33 ± 44.90^ab^	285.95 ± 23.60^a^	263.27 ± 75.47^ab^		0.098	0.042	0.021
**GPx U/mg prot**.								
Duodenum	5.74 ± 1.80	7.97 ± 2.18	9.09 ± 2.31	8.45 ± 1.89	6.41 ± 1.29		0.194	0.836	0.039
Jejunum	7.41 ± 1.73	8.76 ± 2.21	8.92 ± 2.16	10.44 ± 2.15	9.19 ± 2.39		0.576	0.192	0.289
Ileum	28.22 ± 4.97	33.35 ± 1.83	31.38 ± 8.31	30.52 ± 2.76	29.31 ± 1.15		0.670	0.928	0.448
**TAC**, **μmol/g prot**.								
Duodenum	115.56 ± 13.56^b^	128.57 ± 14.30^ab^	133.87 ± 12.73^ab^	151.65 ± 19.32^a^	129.41 ± 9.75^ab^		0.068	0.075	0.048
Jejunum	81.94 ± 4.40	88.27 ± 13.78	100.30 ± 9.23	112.71 ± 15.31	98.18 ± 27.08		0.187	0.056	0.080
Ileum	98.19 ± 15.89	101.69 ± 6.47	118.02 ± 9.27	109.71 ± 3.89	108.30 ± 11.76		0.138	0.157	0.098
**MDA, nmol/mg prot**.								
Duodenum	1.08 ± 0.19^a^	0.73 ± 0.18^b^	0.77 ± 0.16^ab^	0.76 ± 0.20^ab^	0.55 ± 0.07^b^		0.037	0.006	0.021
Jejunum	0.55 ± 0.03^a^	0.30 ± 0.02^b^	0.27 ± 0.06^b^	0.33 ± 0.07^b^	0.41 ± 0.05^ab^		0.033	0.002	0.008
Ileum	0.77 ± 0.12^a^	0.63 ± 0.06^a^	0.41 ± 0.08^b^	0.35 ± 0.07^b^	0.66 ± 0.13^a^		0.001	0.237	0.001
**42 d**								
**CAT U/mg prot**.								
Duodenum	2.56 ± 0.67^c^	2.74 ± 0.59^bc^	4.42 ± 1.03^ab^	4.73 ± 1.02^a^	3.25 ± 0.83^abc^		0.061	0.161	0.060
Jejunum	2.65 ± 0.43	2.94 ± 0.76	2.82 ± 0.67	3.66 ± 0.94	3.35 ± 0.25		0.509	0.134	0.337
Ileum	0.96 ± 0.27	1.34 ± 0.21	1.40 ± 0.23	1.44 ± 0.21	1.32 ± 0.33		0.181	0.084	0.039
**SOD U/mg prot**.								
Duodenum	325.39 ± 85.64^b^	413.26 ± 95.45^ab^	480.48 ± 97.99^a^	513.22 ± 76.83^a^	480.66 ± 58.65^a^		0.076	0.009	0.011
Jejunum	160.38 ± 12.87^b^	163.97 ± 11.10^b^	205.69 ± 7.56^a^	208.75 ± 35.76^a^	165.54 ± 19.60^b^		0.030	0.256	0.035
Ileum	104.24 ± 1.18^c^	141.95 ± 31.31^bc^	146.28 ± 35.33^bc^	181.30 ± 13.77^ab^	227.67 ± 56.39^a^		0.008	0.001	0.001
**GPx U/mg prot**.								
Duodenum	7.31 ± 1.47^b^	8.99 ± 2.42^ab^	9.08 ± 0.53^ab^	10.63 ± 1.63^a^	7.33 ± 1.84^b^		0.090	0.426	0.060
Jejunum	9.90 ± 2.68	10.03 ± 2.87	13.75 ± 2.53	14.81 ± 2.62	11.84 ± 2.39		0.114	0.077	0.090
Ileum	20.10 ± 1.63	24.06 ± 4.98	30.73 ± 7.07	24.26 ± 2.93	22.16 ± 4.56		0.115	0.871	0.061
**TAC**, **μmol/g prot**.								
Duodenum	103.15 ± 13.75^b^	137.88 ± 24.57^a^	149.44 ± 14.21^a^	152.28 ± 24.63^a^	142.22 ± 11.61^a^		0.041	0.027	0.005
Jejunum	120.06 ± 12.50^b^	144.17 ± 14.22^a^	155.42 ± 6.74^a^	143.70 ± 6.34^a^	140.74 ± 8.46^a^		0.011	0.081	0.002
Ileum	63.07 ± 9.01^c^	92.06 ± 17.23^ab^	93.83 ± 14.74^ab^	88.07 ± 10.39^b^	110.61 ± 13.23^a^		0.003	0.001	0.003
**MDA, nmol/mg prot**.								
Duodenum	0.70 ± 0.20	0.68 ± 0.16	0.67 ± 0.14	0.56 ± 0.14	0.61 ± 0.03		0.859	0.327	0.626
Jejunum	0.46 ± 0.01^a^	0.46 ± 0.11^a^	0.33 ± 0.03^b^	0.34 ± 0.01^b^	0.41 ± 0.05^ab^		0.018	0.068	0.031
Ileum	0.46 ± 0.09^a^	0.40 ± 0.07^ab^	0.24 ± 0.03^b^	0.30 ± 0.06^ab^	0.34 ± 0.07^ab^		0.056	0.082	0.020

*AAE, Artemisia annua L. aqueous extract; CAT, catalase; SOD, total superoxide dismutase; GPx, glutathione peroxidase; TAC, total antioxidant capacity; MDA, malondialdehyde*.

*^a−c^Means within a row with different letters differ significantly (p < 0.05), whereas the probability value of 0.05 ≤ p <0.10 was considered as a tendency*.

*Each value is shown as mean ± SD (Data are means for five replicates of eight birds per replicate)*.

### Small Intestine Immune-Related Gene Expression Level

As summarized in Table 7, on day 21, compared with the control group, the AAE groups with the values of 1.5 and 2.0 g/kg significantly decreased the *IL-1*β expression of duodenum (*p* < 0.05); all AAE groups extremely reduced the *IL-1*β expression of ileum (*p* < 0.01), the AAE groups with the values of 1.0–2.0 g/kg remarkably decreased the *IL-6* expression of ileum (*p* < 0.05), and the AAE groups with the values of 1.0 and 2.0 g/kg extremely decreased the *TLR4* expression of ileum (*p* < 0.01). Moreover, with the increase of AAE dose, the gene expression level of duodenal *IL-1*β and *NF-*κ*B/p65*, and the ileal *IL-6* and *TLR4* showed a significant linear reduction effect (*p* < 0.05), and the gene expression of ileal *IL-1*β and *IL-6* showed a quadratic reduction effect (*p* < 0.05). On day 42, compared with the control group, the AAE group with a value of 1.5 g/kg tended to decrease the *IL-1*β expression of duodenum (*p* < 0.10); however, the AAE group with a value of 0.5 g/kg tended to increase the *IL-6* expression of duodenum (*p* < 0.10). In addition, with the increase of AAE dose, the duodenal and jejunal *IL-1*β gene expression showed a linear downward trend (*p* < 0.10), and the duodenal *TLR4*, ileal *IL-1*β, and the jejunal *NF-*κ*B/p65* gene expression showed a quadratic downward trend (*p* < 0.10), while the duodenal *IL-6* gene expression had a quadratic increased effect (*p* < 0.05). The results from a quadratic regression analysis showed that the optimal AAE level that maximized *TLR4* gene expression of the duodenum was 1.2997 g/kg (Table 4).

**Table 7:** Effect of AAE on the expression of small intestinal immune-related genes in broilers.

**Items**	**AAE supplemental level, g/kg**					**p value**		
	**0**	**0.5**	**1.0**	**1.5**	**2.0**	**ANOVA**	**Linear**	**Quadratic**
**21 d**								
**IL-1β**								
Duodenum	1.00 ± 0.00^a^	0.98 ± 0.22^a^	0.99 ± 0.22^a^	0.71 ± 0.18^b^	0.61 ± 0.12^b^	0.010	0.001	0.002
Jejunum	1.00 ± 0.00	0.84 ± 0.22	0.91 ± 0.18	0.89 ± 0.23	0.84 ± 0.14	0.732	0.536	0.558
Ileum	1.00 ± 0.00^a^	0.52 ± 0.08^b^	0.55 ± 0.07^b^	0.50 ± 0.10^b^	0.47 ± 0.06^b^	<0.001	0.001	<0.001
**IL-6**								
Duodenum	1.00 ± 0.00	0.95 ± 0.27	0.86 ± 0.22	0.79 ± 0.20	0.92 ± 0.04	0.563	0.250	0.285
Jejunum	1.00 ± 0.00	0.95 ± 0.27	0.99 ± 0.17	0.91 ± 0.13	0.93 ± 0.19	0.925	0.435	0.742
Ileum	1.00 ± 0.00^a^	0.76 ± 0.21^ab^	0.69 ± 0.17^b^	0.71 ± 0.16^b^	0.64 ± 0.06^b^	0.039	0.010	0.010
**TLR4**								
Duodenum	1.00 ± 0.00	0.90 ± 0.23	0.68 ± 0.13	0.96 ± 0.21	0.86 ± 0.17	0.656	0.537	0.568
Jejunum	1.00 ± 0.00	1.09 ± 0.27	1.06 ± 0.14	1.10 ± 0.22	1.30 ± 0.21	0.936	0.412	0.692
Ileum	1.00 ± 0.00^a^	0.99 ± 0.18^a^	0.76 ± 0.17^bc^	0.94 ± 0.15^ab^	0.59 ± 0.13^c^	0.004	0.004	0.013
**NF-κB/p65**								
Duodenum	1.00 ± 0.00^ab^	1.10 ± 0.13^a^	0.91 ± 0.13^b^	0.91 ± 0.06^b^	0.82 ± 0.19^b^	0.040	0.014	0.035
Jejunum	1.00 ± 0.00	1.03 ± 0.26	0.95 ± 0.21	1.05 ± 0.22	1.05 ± 0.22	0.969	0.724	0.904
Ileum	1.00 ± 0.00	0.88 ± 0.13	1.08 ± 0.28	1.11 ± 0.23	0.97 ± 0.05	0.430	0.510	0.750
**42 d**								
**IL-1β**								
Duodenum	1.00 ± 0.00^a^	1.08 ± 0.22^a^	1.01 ± 0.10^a^	0.72 ± 0.22^b^	0.88 ± 0.18^ab^	0.082	0.068	0.197
Jejunum	1.00 ± 0.00	0.88 ± 0.20	0.92 ± 0.08	0.84 ± 0.24	0.66 ± 0.14	0.128	0.012	0.036
Ileum	1.00 ± 0.00	0.98 ± 0.18	0.92 ± 0.21	0.93 ± 0.07	1.17 ± 0.05	0.144	0.310	0.060
**IL-6**								
Duodenum	1.00 ± 0.00^b^	1.30 ± 0.24^a^	1.23 ± 0.16^ab^	1.27 ± 0.23^ab^	1.03 ± 0.15^b^	0.054	0.752	0.016
Jejunum	1.00 ± 0.00	1.16 ± 0.22	1.04 ± 0.18	1.12 ± 0.26	1.12 ± 0.07	0.764	0.403	0.655
Ileum	1.00 ± 0.00	1.02 ± 0.27	1.20 ± 0.21	1.03 ± 0.13	1.10 ± 0.14	0.600	0.400	0.580
**TLR4**								
Duodenum	1.00 ± 0.00	0.75 ± 0.19	0.63 ± 0.13	0.60 ± 0.09	0.72 ± 0.11	0.119	0.051	0.020
Jejunum	1.00 ± 0.00	1.42 ± 0.30	1.03 ± 0.20	0.99 ± 0.13	1.16 ± 0.15	0.585	0.913	0.925
Ileum	1.00 ± 0.00	0.85 ± 0.27	1.10 ± 0.21	1.14 ± 0.18	1.06 ± 0.13	0.720	0.427	0.729
**NF-κB/p65**								
Duodenum	1.00 ± 0.00	1.07 ± 0.15	1.24 ± 0.28	1.16 ± 0.07	1.06 ± 0.23	0.308	0.287	0.117
Jejunum	1.00 ± 0.00^ab^	0.95 ± 0.12^ab^	1.09 ± 0.19^a^	0.92 ± 0.02^ab^	0.82 ± 0.08^b^	0.065	0.085	0.069
Ileum	1.00 ± 0.00	0.93 ± 0.23	1.15 ± 0.20	1.05 ± 0.26	0.99 ± 0.07	0.752	0.770	0.720

*AAE, Artemisia annua L. aqueous extract; IL-1β, interleukin 1 beta; IL-6, interleukin 6; TLR4, toll like receptor 4; NF-κB /p65, nuclear factor kappa B/p65*.

*^a−c^Means within a row with different letters differ significantly (p < 0.05), whereas the probability value of 0.05 ≤ p < 0.10 was considered as a tendency*.

*Each value is shown as mean ± SD (Data are means for five replicates of eight birds per replicate)*.

### Small Intestine Antioxidant Related Gene Expression Level

As shown in Table 8, on day 21, compared with the control group, *CAT* gene expression in duodenum and ileum of the AAE groups with the values of 1.0 and 1.5 g/kg, and jejunum of AAE groups with the values of 0.5, 1.0, and 2.0 g/kg was significantly increased (*p* < 0.05, *p* < 0.01, *p* < 0.01). And the AAE group with a value of 1.0 g/kg significantly increased the duodenal *SOD* and *Nrf2* gene expression (*p* < 0.05), and the ileal *Keap1* gene expression in all AAE groups was lower (*p* < 0.05). Moreover, with the increase of AAE dose, the jejunal *SOD* and *GPx* gene expression showed a linear upward trend (*p* < 0.10), and the gene expression of *CAT* in the three parts of small intestine showed a significant quadratic increased effect (*p* < 0.01). The jejunal *HO-1* gene expression showed a linear increased effect (*p* < 0.01). The duodenal *Nrf2* gene expression showed a quadratic upward trend (*p* < 0.10). The ileal *Keap1* gene expression showed a quadratic reduction effect (*p* < 0.01). According to a quadratic regression analysis, the minimum response for *Keap1* gene expression of the ileum was observed at AAE level of 1.3497 g/kg (Table 4). Additionally, on day 42, compared with the control group, *SOD* gene expression in duodenum of AAE group with a value of 1.5 g/kg, jejunum of AAE group with a value of 2.0 g/kg, and ileum of AAE group with a value of 1.0 g/kg was increased (*p* < 0.05, *p* < 0.10, *p* < 0.05). And the jejunal *HO-1* gene expression in the AAE group with the values of 1.5 and 2.0 g/kg was significantly increased (*p* < 0.01). The ileal *HO-1* and *Nrf2* gene expression in all AAE groups was significantly increased (*p* < 0.05). The jejunal *Keap1* gene expression in the AAE groups with the values of 1.0 and 1.5 g/kg was significantly reduced (*p* < 0.05). Besides, with the increase of AAE dose, the duodenal and jejunal *SOD* gene expression showed a linear increased effect (*p* < 0.10; *p* < 0.05), and the jejunal and ileal *HO-1* and *Nrf2* gene expression showed a linear increased effect (*p* < 0.05); moreover, the duodenal *Nrf2* and jejunal *Keap1*, ileal *SOD* and *GPx* gene expression showed a quadratic increased effect (*p* < 0.05, *p* < 0.01, *p* < 0.10, *p* < 0.10). As shown in Table 4, for ileum *CAT* gene expression, the optimal AAE level was 1.1614 g/kg.

**Table 8:** Effect of AAE on the expression of small intestinal antioxidant-related genes in broilers.

**Items**	**AAE supplemental level, g/kg**					***p*** **-value**		
	**0**	**0.5**	**1.0**	**1.5**	**2.0**	**ANOVA**	**Linear**	**Quadratic**
**21 d**								
**CAT**								
Duodenum	1.00 ± 0.00^b^	1.17 ± 0.08^ab^	1.46 ± 0.17^a^	1.44 ± 0.26^a^	1.30 ± 0.24^ab^	0.048	0.022	0.009
Jejunum	1.00 ± 0.00^c^	1.41 ± 0.11^b^	1.89 ± 0.37^a^	1.38 ± 0.12^bc^	1.52 ± 0.32^ab^	0.003	0.037	0.005
Ileum	1.00 ± 0.00^b^	1.28 ± 0.28^b^	1.90 ± 0.30^a^	1.78 ± 0.14^a^	1.28 ± 0.25^b^	<0.001	0.030	0.001
**SOD**								
Duodenum	1.00 ± 0.00^b^	0.79 ± 0.09^b^	1.53 ± 0.30^a^	1.20 ± 0.28^ab^	1.03 ± 0.02^b^	0.033	0.458	0.207
Jejunum	1.00 ± 0.00	1.05 ± 0.26	1.35 ± 0.35	1.31 ± 0.26	1.30 ± 0.06	0.322	0.068	0.136
Ileum	1.00 ± 0.00	0.96 ± 0.22	1.05 ± 0.09	0.94 ± 0.08	0.82 ± 0.18	0.306	0.110	0.140
**GPx**								
Duodenum	1.00 ± 0.00	0.95 ± 0.25	0.99 ± 0.16	1.04 ± 0.27	0.80 ± 0.14	0.535	0.347	0.419
Jejunum	1.00 ± 0.00	1.23 ± 0.11	0.99 ± 0.19	1.31 ± 0.30	1.27 ± 0.31	0.126	0.066	0.194
Ileum	1.00 ± 0.00	0.89 ± 0.23	1.08 ± 0.25	0.81 ± 0.10	0.75 ± 0.18	0.243	0.100	0.200
**HO-1**								
Duodenum	1.00 ± 0.00	1.37 ± 0.26	1.84 ± 0.31	1.31 ± 0.17	1.51 ± 0.12	0.130	0.177	0.106
Jejunum	1.00 ± 0.00^ab^	0.97 ± 0.17^b^	0.99 ± 0.26^ab^	1.45 ± 0.30^a^	1.41 ± 0.29^ab^	0.052	0.009	0.027
Ileum	1.00 ± 0.00	0.88 ± 0.21	0.95 ± 0.14	0.95 ± 0.07	0.98 ± 0.19	0.990	0.994	0.924
**Keap1**								
Duodenum	1.00 ± 0.00	1.00 ± 0.22	1.08 ± 0.13	0.99 ± 0.25	0.91 ± 0.12	0.969	0.736	0.800
Jejunum	1.00 ± 0.00	1.11 ± 0.27	1.13 ± 0.15	1.41 ± 0.29	0.76 ± 0.09	0.262	0.944	0.288
Ileum	1.00 ± 0.00^a^	0.76 ± 0.08^b^	0.74 ± 0.18^b^	0.60 ± 0.10^b^	0.75 ± 0.14^b^	0.019	0.010	0.004
**Nrf2**								
Duodenum	1.00 ± 0.00^b^	1.07 ± 0.24^b^	2.75 ± 0.23^a^	2.02 ± 0.08^ab^	1.71 ± 0.31^b^	0.023	0.134	0.099
Jejunum	1.00 ± 0.00	0.97 ± 0.26	0.99 ± 0.27	1.09 ± 0.17	1.21 ± 0.23	0.823	0.284	0.447
Ileum	1.00 ± 0.00	0.93 ± 0.18	1.29 ± 0.23	1.29 ± 0.15	1.26 ± 0.27	0.613	0.160	0.363
**42 d**								
**CAT**								
Duodenum	1.00 ± 0.00	0.94 ± 0.23	0.93 ± 0.10	1.02 ± 0.06	0.98 ± 0.24	0.944	0.980	0.905
Jejunum	1.00 ± 0.00	0.99 ± 0.23	1.03 ± 0.13	0.89 ± 0.17	1.07 ± 0.22	0.836	0.854	0.872
Ileum	1.00 ± 0.00	1.02 ± 0.25	1.03 ± 0.17	1.09 ± 0.23	0.99 ± 0.18	0.982	0.860	0.910
**SOD**								
Duodenum	1.00 ± 0.00^bc^	0.91 ± 0.07^c^	1.17 ± 0.06^ab^	1.22 ± 0.07^a^	1.09 ± 0.23^abc^	0.026	0.057	0.091
Jejunum	1.00 ± 0.00^bc^	0.88 ± 0.21^c^	1.13 ± 0.13^ab^	1.08 ± 0.14^abc^	1.25 ± 0.19^a^	0.052	0.018	0.049
Ileum	1.00 ± 0.00^b^	1.23 ± 0.11^ab^	1.54 ± 0.33^a^	1.13 ± 0.21^b^	1.13 ± 0.18^b^	0.047	0.470	0.060
**GPx**								
Duodenum	1.00 ± 0.00	1.10 ± 0.22	1.12 ± 0.23	1.05 ± 0.19	1.21 ± 0.28	0.771	0.264	0.546
Jejunum	1.00 ± 0.00	1.05 ± 0.12	1.34 ± 0.32	1.18 ± 0.25	1.06 ± 0.26	0.315	0.505	0.194
Ileum	1.00 ± 0.00	0.98 ± 0.27	0.92 ± 0.23	0.93 ± 0.17	1.17 ± 0.26	0.159	0.310	0.060
**HO-1**								
Duodenum	1.00 ± 0.00	1.05 ± 0.21	1.23 ± 0.30	1.12 ± 0.07	0.92 ± 0.25	0.582	0.979	0.298
Jejunum	1.00 ± 0.00^c^	1.25 ± 0.22^bc^	1.43 ± 0.31^bc^	1.65 ± 0.29^b^	2.31 ± 0.22^a^	<0.001	<0.001	<0.001
Ileum	1.00 ± 0.00^b^	2.05 ± 0.39^a^	2.12 ± 0.40^a^	2.70 ± 0.21^a^	2.73 ± 0.17^a^	<0.001	<0.001	<0.001
**Keap1**								
Duodenum	1.00 ± 0.00	0.99 ± 0.18	1.03 ± 0.11	1.14 ± 0.20	1.08 ± 0.22	0.774	0.289	0.575
Jejunum	1.00 ± 0.00^a^	0.78 ± 0.17^ab^	0.70 ± 0.12^b^	0.52 ± 0.10^b^	0.81 ± 0.18^ab^	0.013	0.025	0.005
Ileum	1.00 ± 0.00	0.93 ± 0.23	1.02 ± 0.15	0.77 ± 0.14	1.35 ± 0.27	0.181	0.265	0.166
**Nrf2**								
Duodenum	1.00 ± 0.00	1.32 ± 0.18	1.24 ± 0.26	0.92 ± 0.06	0.83 ± 0.14	0.358	0.113	0.044
Jejunum	1.00 ± 0.00^ab^	0.82 ± 0.21^b^	1.34 ± 0.32^ab^	1.45 ± 0.26^a^	1.26 ± 0.18^ab^	0.066	0.038	0.103
Ileum	1.00 ± 0.00^b^	1.56 ± 0.31^a^	1.54 ± 0.22^a^	1.52 ± 0.16^a^	1.70 ± 0.30^a^	0.040	0.007	0.015

*AAE, Artemisia annua L. aqueous extract; CAT, catalase; SOD, total superoxide dismutase; GPx, glutathione peroxidase; HO-1, heme oxygenase-1; Keap1, Kelch-like ECH-associated protein-1; Nrf2, nuclear factor-erythroid 2-related factor 2*.

*^a−c^Means within a row with different letters differ significantly (p < 0.05), whereas the probability value of 0.05 ≤ p < 0.10 was considered as a tendency*.

*Each value is shown as mean ± SD (Data are means for five replicates of eight birds per replicate)*.

## Discussion

To the best of our knowledge, *A. annua* and its derivatives have a variety of biological functions, and show an excellent anti-inflammatory and immunomodulatory activity in the intestinal tract of animals (33). In the present study, the content of intestinal pro-inflammatory cytokines, including IL-1β and IL-6, decreased in a dose-dependent fashion with the increase of dietary AAE, suggesting a greater improvement on the anti-inflammatory level of the intestine in broilers. Similar results were observed by Niu et al. (25) who found that diet supplemented with enzymatically treated *A. annua* markedly decreased the content of IL-1β and IL-6 in intestinal mucosa of weaned pigs. Furthermore, studies found that the concentration of pro-inflammatory cytokines IL-1β and IL-6 was reduced, which was related to the content of immunoglobulin in the intestinal mucosa, and increased immunoglobulin in the small intestine promoted efficient prevention of intestinal inflammatory conditions (34). Our study found that the content of secretory IgA (sIgA), IgG, IgM in the small intestine of broilers increased with the increase of dietary AAE. Similarly, Niu et al. (25) reported that a diet supplemented with enzymatically treated *A. annua* increased the content of sIgA and IgG in the jejunum and ileum mucosa of weaned pigs. In poultry, three classes of immunoglobulins bind antigens specifically and remove them through precipitation and phagocytosis. Here, in the current study, dietary AAE supplementation decreased the gene expression of *IL-1*β and *IL-6* in the small intestinal mucosa of broilers, which was consistent with the decrease in their content. A previous study showed that the gene expression of *IL-1*β and *IL-6* in broiler chickens was regulated by the NF-κB signaling pathway, and the NF-κB signaling pathway was activated by the transmembrane signal transporter TLR4 (35). Our study demonstrated that the ileal *TLR4* gene expression showed an extremely significant linear reduction effect with the increase of AAE dose on day 21. Furthermore, the duodenal *NF-*κ*B/p65* gene expression showed a significant linear reduction effect with the increase of AAE dose. Similarly, Zhang et al. (36) found that the nuclear translocation of p65 was also significantly inhibited by Artemargyinolide E (a new sesquiterpene lactone from *Artemisia argyi*) *in vitro*. Moreover, a previous study showed that inflammation could be regulated *via* the TLR-4/NF-κB signaling pathway in mice and broilers (37, 38). Thus, based on the results of this study, we preliminarily speculated that AAE could reduce the content and gene expression level of IL-1β and IL-6 in the intestinal mucosa of broilers by regulating the TLR4/NF-κB signaling pathway. The reason might be that *A. annua* aqueous extract contains bioactive components (polysaccharides, polyphenols, flavonoids) that play an immunoregulatory role (18, 27), and previous studies showed that polysaccharides in *Artemisia* could regulate the immune function of broilers through the TLR4/NF-κB signaling pathway (39). However, the specific mechanism of dietary AAE regulating intestinal immune function of broilers needs further study.

*A. annua* is rich in a variety of bioactive substances, including flavonoids, polysaccharides, coumarins, and sesquiterpenes, which have strong antioxidant properties (39, 40). And previous studies reported that dietary *A. annua* enhanced the antioxidant capacity of plasma in laying hens (22). Besides, Song et al. (26) reported that diets added with enzymatically treated *A. annua* improved the activity of CAT and SOD, and decreased the MDA concentration in small intestinal of broilers under the thermoneutral condition, indicating that *A. annua* could improve the antioxidant capacity of the body. Our study was conducted in a conventional feeding pattern, and we found that the activity of CAT, SOD, and GPx in the small intestinal increased quadratically with the increase of dietary AAE, and also decreased MDA concentration. This was consistent with our previous research results, which reported that AAE increased the activity of CAT, SOD, GPx, and TAC, and reduced the concentration of MDA in serum, hepatic, and spleen of broilers (40). Moreover, the AAE showed strong antioxidant activity in the small intestine and also upregulated the expression of antioxidant-related genes (*SOD, CAT, GPx, HO-1, Nrf2*) in small intestine of broilers in the present study. Nrf2 regulates the expression of antioxidant response element (ARE)-driven antioxidants and phase II detoxifying enzymes such as SOD, CAT, GPx, and HO-1, which exhibit cytoprotective effects against oxidative stress in various cells. *Heme oxygenase-1* (*HO-1*), the downstream gene of Nrf2 pathway, can reduce oxidative injury by catalyzing heme and the subsequent production of bioactive metabolites. In keeping with our findings, Xing et al. (41) reported that *Artemisia ordosica* polysaccharide could improve the antioxidant capacity of rats by upregulating the gene expression level of *SOD, CAT*, and *GPx*. Simultaneously, another study showed that *Artemisia ordosica* polysaccharide could increase the activity of the antioxidant enzyme in liver of broilers and improve antioxidant status through the Nrf2/Keap1 pathway (38). Moreover, some studies manifested that *A. annua* extract upregulated the mRNA expression of *GPx* and *SOD* in small intestine of broilers, which was consistent with the results of antioxidant-related enzyme activity, meanwhile, and the mRNA expression of *Nfe2l2* and *Hmox1* was following the protein expression of Nrf2 and HO-1, which indicated that *A. annua* extract improved intestinal antioxidant capacity by activating the related mRNA and protein expression of the Nrf2/ARE-mediated pathway (26). Thus, we preliminarily speculated that AAE regulated the activity and gene expression of *CAT, SOD*, and *GPx* by upregulating *Nrf2* and *HO-1* gene expressions. In the present study, dietary AAE enhanced the activity of small intestinal TAC, SOD, CAT, and GPx in broilers, and the changes of these parameters might be related to the mechanism of the antioxidant system. We preliminarily speculated that the reason why AAE could improve the antioxidant function of the body might be related to the antioxidant activity of flavonoids and polyphenols contained in AAE (40). This is due to the fact that a variety of bioactive substances have strong free radical scavenging activity, and the stronger free radical scavenging ability is positively correlated with the antioxidant ability. However, the specific antioxidant mechanism of AAE needs further investigation.

## Conclusion

The inclusion of AAE in the diet improved the intestinal immunoglobulins, inflammatory cytokines, and related mRNA expressions through the NF-κB signaling pathway. Moreover, AAE could regulate antioxidant enzyme activity and relative mRNA expressions in intestinal mucous through Nrf2 signaling pathway of broilers. The optimal level of AAE supplementation in the diet was 1.12–1.38 g/kg.

## Data Availability Statement

The datasets presented in this study can be found in online repositories. The names of the repository/repositories and accession number(s) can be found in the article/supplementary material.

## Ethics Statement

The animal study was reviewed and approved by GB/T 35892-2018. Written informed consent was obtained from the owners for the participation of their animals in this study.

## Author Contributions

Conceptualization: SG and BS. Validation, supervision, project administration, and funding acquisition: BS. Formal analysis: SG and JM. Investigation and data curation: SG. Resources: YXu, SY, and BS. Writing—Original draft preparation: SG and LS. Writing—Review & editing: SG and LZ. Visualization: SG and YXi. All authors contributed to the article and approved the submitted version.

## Conflict of Interest

The authors declare that the research was conducted in the absence of any commercial or financial relationships that could be construed as a potential conflict of interest.

## Publisher's Note

All claims expressed in this article are solely those of the authors and do not necessarily represent those of their affiliated organizations, or those of the publisher, the editors and the reviewers. Any product that may be evaluated in this article, or claim that may be made by its manufacturer, is not guaranteed or endorsed by the publisher.
